# Onvansertib exhibits anti-proliferative and anti-invasive effects in endometrial cancer

**DOI:** 10.3389/fphar.2025.1545038

**Published:** 2025-03-17

**Authors:** Nikita Sinha, Xiaochang Shen, Jennifer Haag, Shuning Chen, Haomeng Zhang, Catherine John, Wenchuan Sun, Michael Emanuele, Chunxiao Zhou, Victoria Bae-Jump

**Affiliations:** ^1^ Division of Gynecologic Oncology, University of North Carolina at Chapel Hill, Chapel Hill, NC, United States; ^2^ Department of Gynecology, Beijing Obstetrics and Gynecology Hospital, Beijing Maternal and Child Healthcare Hospital, Capital Medical University, Beijing, China; ^3^ Department of Pharmacology, University of North Carolina at Chapel Hill, Chapel Hill, NC, United States; ^4^ Lineberger Comprehensive Cancer Center, University of North Carolina at Chapel Hill, Chapel Hill, NC, United States

**Keywords:** onvansertib, polo-like kinase 1, endometrial cancer, cell proliferation, invasion, apoptosis

## Abstract

Polo-like kinase 1 (Plk1) is widely recognized as an oncogene that promotes cell proliferation by regulating cell division, DNA damage response, and genome stability and has been shown to be overexpressed in many cancers, including endometrial cancer. Targeting Plk1 by onvansertib has been shown to have anti-tumor activity in pre-clinical models of multiple cancers and is currently being evaluated in phase 1 and 2 clinical trials in cancer patients. In this study, we evaluated the potential anti-tumorigenic effects of onvansertib in endometrial cancer cells and the LKB1^fl/fl^ p53^fl/fl^ mouse model of endometrial cancer. Onvansertib inhibited cellular proliferation, caused G2 phase arrest, induced cellular stress and apoptosis, and inhibited cellular migration and invasion in endometrial cancer cells. Combined treatment with onvansertib and paclitaxel led to synergistic inhibition of cell proliferation. Onvansertib treatment for 4 weeks significantly reduced tumor growth in LKB1^fl/fl^p53^fl/fl^ mice. Given these promising pre-clinical results, further studies are needed to evaluate the clinical translatability of onvansertib combined with paclitaxel as an effective treatment for endometrial cancer.

## Introduction

Endometrial cancer (EC) is one of the few cancers for which incidence and mortality continue to rise steadily, attributed partly to the worsening obesity epidemic in the United States. It is the fourth most common cancer in women, and in 2024, it is predicted that 67,880 new cancers of endometrial cancer will be diagnosed and 13,250 women will die from endometrial cancer (Siegel et al., 2024). While endometrial cancer is typically detected early and curable with surgery, a significant portion of early-stage patients may recur. Advanced or recurrent EC carries a much worse prognosis, with survival rates of less than 20% (Cao et al., 2023). Only a handful of effective treatment strategies are available for advanced-stage and recurrent EC. Thus, additional novel therapies are crucially necessary to reverse the worsening mortality trends (Kuhn et al., 2023; Karpel et al., 2023). With an improved understanding of the molecular drivers of EC, the door has opened to the use of innovative targeted therapies for EC management.

Polo-like kinase 1 (Plk1), a member of the Polo-like kinase family, is an evolutionarily conserved Ser/Thr kinase involved in controlling cell division, modulating DNA damage response, and maintaining genome stability (Degenhardt and Lampkin, 2010; Gheghiani and Fu, 2023). Numerous studies, including The Cancer Genome Atlas (TCGA) cohort analysis, have shown that overexpression of Plk1 is present in multiple types of cancer and is closely associated with aggressive behavior and poor prognosis (Gheghiani and Fu, 2023; Weng et al., 2016; Liu et al., 2017). In patients with EC, Plk1 mRNA expression was found to be significantly elevated, showing a 21-fold increase compared to the normal endometrium. Additionally, the overexpression of Plk1 protein was closely linked to higher histological grade and invasiveness of EC (8, 9). Elevated Plk1 activity significantly inhibits cell death pathways through the processes of autophagy and apoptosis, thereby promoting cancer cell proliferation (Chiappa et al., 2022; Matthess et al., 2014). Targeting Plk1 by siRNA or small molecule inhibitors effectively reduces cell proliferation, causes cell cycle arrest, induces apoptosis, inhibits cell invasion, and increases the sensitivity to chemotherapeutic agents through β-catenin/c-Myc, AKT/mTOR, and ERK Pathways in multiple pre-clinical models of cancer (Matthess et al., 2014; Song et al., 2018; Fu and Wen, 2017; Wang et al., 2023; Choi et al., 2023). Over the past decade, several Plk1 inhibitors have entered into clinical trials for many different tumor types to evaluate their anti-tumorigenic activity and safety, and these Plk1 inhibitors exhibit promising efficacy and a good safety profile in many of these cancer types. Clinical trials of Plk1 in combination with other chemotherapeutic agents have also shown good tolerability and significant clinical activity in hematological malignancies (Liu et al., 2017; Su et al., 2022; Wei et al., 2023). More clinical trials evaluating combination treatments in cancer patients are underway, including small cell lung cancer, breast cancer, and prostate cancer.

Onvansertib is a novel oral, highly selective adenosine triphosphate (ATP) competitive Plk1 inhibitor that has demonstrated anti-tumorigenic activity in multiple types of solid tumors and hematologic malignancies, including AML, medulloblastoma, ovarian cancer, breast cancer, and colon cancer (Wang et al., 2022; Affatato et al., 2020; Stebbing and Bullock, 2024a). Recent pre-clinical studies have found that onvansertib in combination with paclitaxel also exhibits synergistic effects in breast and ovarian cancer and improves cisplatin resistance in lung and head and neck squamous cell carcinomas (Affatato et al., 2022; Hagege et al., 2021). Several phase 1b clinical trials confirm that onvansertib shows promising clinical activity in combination with other chemotherapeutic agents in patients with AML or colorectal cancer and appears to be overall well-tolerated (Ahn et al., 2024; Zeidan et al., 2020; Croucher et al., 2023). Considering the encouraging anti-tumorigenic activity of onvansertib in solid tumors and the clinical relevance of Plk1 overexpression in EC, we aimed to investigate the effect of onvansertib in human EC cell lines and endometrioid EC transgenic mouse models.

## Materials and methods

### Cell culture and reagents

Four human endometroid EC cell lines, KLE, EC-023, HEC-1B, and Ishikawa, were used in this study. KLE cells were cultured in DMEM/F12 medium with 10% fetal bovine serum (FBS). EC-023 cells were kindly provided by Dr. Jay Gertz from the University of Utah and cultured in RPMI 1640 medium supplemented with 10% FBS. HEC-1B cells were cultured in Mccoy’s 5A supplemented with 10% FBS. Ishikawa cells were cultured in MEM medium supplemented with 5% FBS. All media was supplemented with 2 mM L-glutamine, 100 U/mL penicillin, and 100 µg/mL streptomycin. Cells were cultured in an incubator under 5% CO_2_ at 37°C. Onvansertib was purchased from MCE (Cat# HY-15828) (Monmouth Junction, NJ, United States). All antibodies were purchased from Cell Signaling Technology (Beverly, MA) and Abclonal Science (Woburn, MA, United States). All other chemicals were purchased from Thermo Fisher Scientific (Waltham, MA), Sigma, and MedChemExpress (Monmouth Junction, NJ).

### Cell proliferation assay

The KLE and EC-023 cells were plated in 96-well plates with a concentration of 4,000 cells/well. After plating for 24 h, the cells were treated with 0.1–500 nM of onvansertib for 72 h 5 μL of 3-(4,5-Dimethyl-2-thiazolyl)-2,5-diphenyl-2H-tetrazolium Bromide (MTT, 5 mg/mL) was added into each well, followed by incubation for 1 h. After mixing 100 µL dimethyl sulfoxide (DMSO) into each well, MTT absorbance was obtained using a microplate reader (Tecan, Morrisville, NC) at a wavelength of 562 nm. The effect of onvansertib on cell proliferation was calculated as a percentage of control, and the IC50 was calculated using the AAT Bioquest IC50 calculator (Pleasanton, CA). The Bliss independence model was used to calculate the synergistic effect as CI < 1 (synergistic), CI = 1 (additive), or CI > 1 (antagonistic). Each experiment was performed with three biological replicates and four technical replicates per condition.

### Colony formation assay

The KLE and EC-023 cells were cultured overnight in 6-well plates with a concentration of 400–800 cells/well. The cells were then treated with 10, 25, and 50 nM of onvansertib or the control (DMSO) for 48 h. The cells were cultured for 10–14 days with fresh medium changes every 3–4 days. After adding methanol, the cells were stained with 0.5% crystal violet buffer for 30 min. The clones were imaged and quantified using ImageJ software (V1.8.0) (National Institutes of Health, Bethesda, MD). Each experiment was performed with three biological replicates and three technical replicates per condition.

### Cell cycle assay

The KLE and EC-023 cells were plated in 6-well plates overnight and then treated with 10, 25, and 50 nM of onvansertib for 24 h. Cells were collected, washed with PBS, and resuspended in ice-cold 90% methanol for 30 min. The methanol was removed, and the cells were resuspended in 100 µL RNase A solution with propidium iodide (2 mg/mL) at room temperature in dark conditions for 20 min. The profile of cell cycle progression was obtained using Cellometer (Nexcelom, Lawrence, MA). FCS4 express software (Molecular Devices, Sunnyvale, CA) was used to analyze the distribution of the cell cycle. The experiment was repeated three times. Data were derived from three independent biological experiments.

### Cleaved caspase 3 ELISA assay

The KLE and EC-023 cells were cultured in 6-well plates at a concentration of 2 × 10^5^ cells/mL overnight and then treated with 10, 25, and 50 nM of onvansertib for 14 h. After washing each well with PBS, 150 µL of caspase lysis buffer was added to each well, and the collection was centrifuged for 15 min. The concentration of protein in each well was detected using a BCA kit (Thermo Fisher). Equal amounts of protein and reaction buffer with caspase three substrate were added to a new black 96-well plate at 37°C for 20 min. The fluorescence intensity for cleaved caspase 3 (Ex/Em = 341/441) was measured using a Tecan microplate reader. The experiment was repeated three times. Each experiment was performed with three biological replicates and three technical replicates per condition.

### Reactive oxygen species (ROS) assay

KLE and EC-023 cells were cultured overnight in 96-well plates with a concentration of 1 × 10^4^ cells/well. They were then exposed to 10, 25, and 50 nM of onvansertib for 6 h 15 μM DCFH-DA was added into each well, and the plate was incubated at 37°C for 30 min. The ROS production was obtained by detecting the fluorescence intensity at Ex/Em = 485/526 nm using a Tecan microplate reader. All experiments were performed at least three times for consistency. Each experiment was performed with three biological replicates and four technical replicates per condition.

### Mitochondrial membrane potential assay

Mitochondrial membrane potential was determined using a specific fluorescent probe for JC-1 (AAT Bioquest, Sunnyvale, CA, United States). KLE and EC-023 cells were seeded in 96-well plates overnight and then treated with 10, 25, and 50 nM of onvansertib for 6 h. Cells were then treated with 2 µM JC-1 for 30 min at 37°C. The levels of fluorescent probes were measured using a Tecan plate reader. The wavelength of fluorescence intensity for JC-1 was Ex/Em = 535/590 nm (red) and Ex/Em = 485/535 nm (green). Each experiment was performed with three biological replicates and four technical replicates per condition.

### Adhesion assay

96-well plates were coated with 100 µL of laminin-1 (10 µg/mL) and incubated overnight at 4°C. The plate was rinsed twice with PBS, and then 100 µL of blocking buffer was added into each well for 45 min at 37°C. 1.5 × 10^4^ cells of KLE and EC-023 were added to each well, followed by 10, 25, and 50 nM of onvansertib and incubation at 37°C for 2 hours. The plate was washed with PBS to remove nonadherent cells. 100 μL of 5% glutaraldehyde was then added to fix cells for 30 min. Finally, the cells were treated with 100 µL of 0.1% crystal violet for 15 min and dissolved in 100 µL of 10% acetic acid. The absorbance was obtained at a wavelength of 575 nm using a Tecan microplate reader. Each experiment was performed with three biological replicates and four technical replicates per condition.

### Wound healing assay

The KLE and EC-023 cells were cultured at a concentration of 2 × 10^5^ cells/mL in 6-well plates overnight. A 200 µL pipette tip was used to draw straight lines in each well, and the cells were treated with 10, 25, and 50 nM of onvansertib. Photos were taken 24–48 h after treatment, and the width of the wound was measured with ImageJ software (National Institutes of Health, Bethesda, MD). Each experiment was performed with three biological replicates and three technical replicates per condition.

### Western immunoblotting

The KLE and EC-023 cells were exposed to 10, 25, and 50 nM of onvansertib, and then the total protein was harvested with radioimmunoprecipitation assay buffer (RIPA buffer, Thermo Fisher). The protein concentration was determined via BCA assay. Protein was separated using electrophoresis with 10%–12% acrylamide gel and transferred onto a PVDF membrane. The membranes were incubated with primary antibody (1:1000) overnight at 4°C. The appropriate secondary antibody was incubated with membranes for 1 hour at room temperature. Proteins were visualized using SuperSignal West Pico Substrate (Thermo Scientific) via the ChemiDoc image system (Bio-Rad, Hercules, CA). Data were derived from three independent biological experiments.

### *Lkb1* ^*fl/fl*^ *p53* ^*fl/fl*^ transgenic mouse model of EC

The *Lkb1* ^*fl/fl*^ *p53* ^*fl/fl*^ transgenic mouse model created in our lab was used to investigate the effect of onvansertib on tumor growth *in vivo*. After AdCre induction, the model progressed to complex atypical hyperplasia at 4–5 weeks and to early invasive cancer at 6–7 weeks. H&E staining showed well-differentiated adenocarcinoma with wild-type p53 and Lkb1 inactivation (Guo et al., 2019). This model better represents the progression of human endometrial cancer under conditions of obesity and leanness and has recently been used in multiple translational studies of EC (Zhao et al., 2014a; Zhao et al., 2014b; Kong et al., 2024). The mice were housed at our animal facility with a 12-h light, 12-h dark cycle, and allowed free access to food and water. Recombinant adenovirus Ad5-CMV-Cre (AdCre, Transfer Vector Core, University of Iowa) at a titer of 2.5 × 10^11^ P.F.U was used for intrauterine injection and tumor induction. Eight-week-old female mice were injected with 5 µL of Ad-Cre in the left uterine horn. After 9 weeks following injection, the mice were randomly divided into two groups: control and onvansertib treatment groups (14 mice per group), and treated with onvansertib (25 mg/kg, 100 µL per mouse for oral gavage, daily) or the vehicle (100 μL, 0.5% methylcellulose, daily) for 4 weeks, with the body weight weighed every week throughout the treatment. All mice were sacrificed by CO_2_ asphyxiation after 4 weeks of treatment. Endometrial tumors were collected and weighed, with one-half of the endometrial tumors fixed in 10% neutral-buffered formalin and paraffin-embedded, and the remaining half snap-frozen and stored at −80°C. Animal experiments were approved by the Institutional Animal Care and Use Committee (IACUC) of the University of North Carolina at Chapel Hill (UNC-CH) (protocol # 21–209).

### Immunohistochemistry (IHC) of endometrial tumors

The endometrial tumors (6 samples per group) obtained from control and onvansertib groups were fixed with formalin for 48 h, embedded with paraffin, and cut into sections (4 μM) at the Animal Histopathology Core Facility at UNC-CH. The slides were deparaffinized with xylene and hydrated with gradually diluted ethanol. After boiling the slides for 3 min in a pressure cooker with appropriate antigen retrieval buffer, the slides were soaked in cold water for 10 min and treated with 3% hydrogen peroxide to block endogenous peroxidase activity. After that, protein block solution (Dako, Agilent Technologies, Santa Clara, CA) was used to block the slides for 30 min at room temperature and then the slides were incubated with primary antibodies of Ki-67 (1:300), Bcl-xL (1: 1200), Plk1 (1:200) overnight at 4°C. The slides were then washed and incubated with appropriate secondary antibodies (Vector Labs, Burlingame, CA) at room temperature for 1 h. ABC Substrate System (Vector Labs) was used for the color reaction, 3,3′-Diaminobenzidine (DAB) was used as a chromogen, and Mayer’s hematoxylin was used for counterstaining. Individual slides were scanned using Motic (Feasterville, PA), and digital images were analyzed for target protein expression using ImagePro software (Vista, CA).

### Statistical analysis

All data were combined from three independent assays into a mean ± SD. Both Student’s T-tests and one-way ANOVA tests were used in this study. The statistical analysis was completed using GraphPad Prism 10 (La Jolla, CA, United States). All tests were two-sided with p < 0.05 considered significant.

## Results

### Effect of onvansertib on cell proliferation in EC cells

**FIGURE 1:**
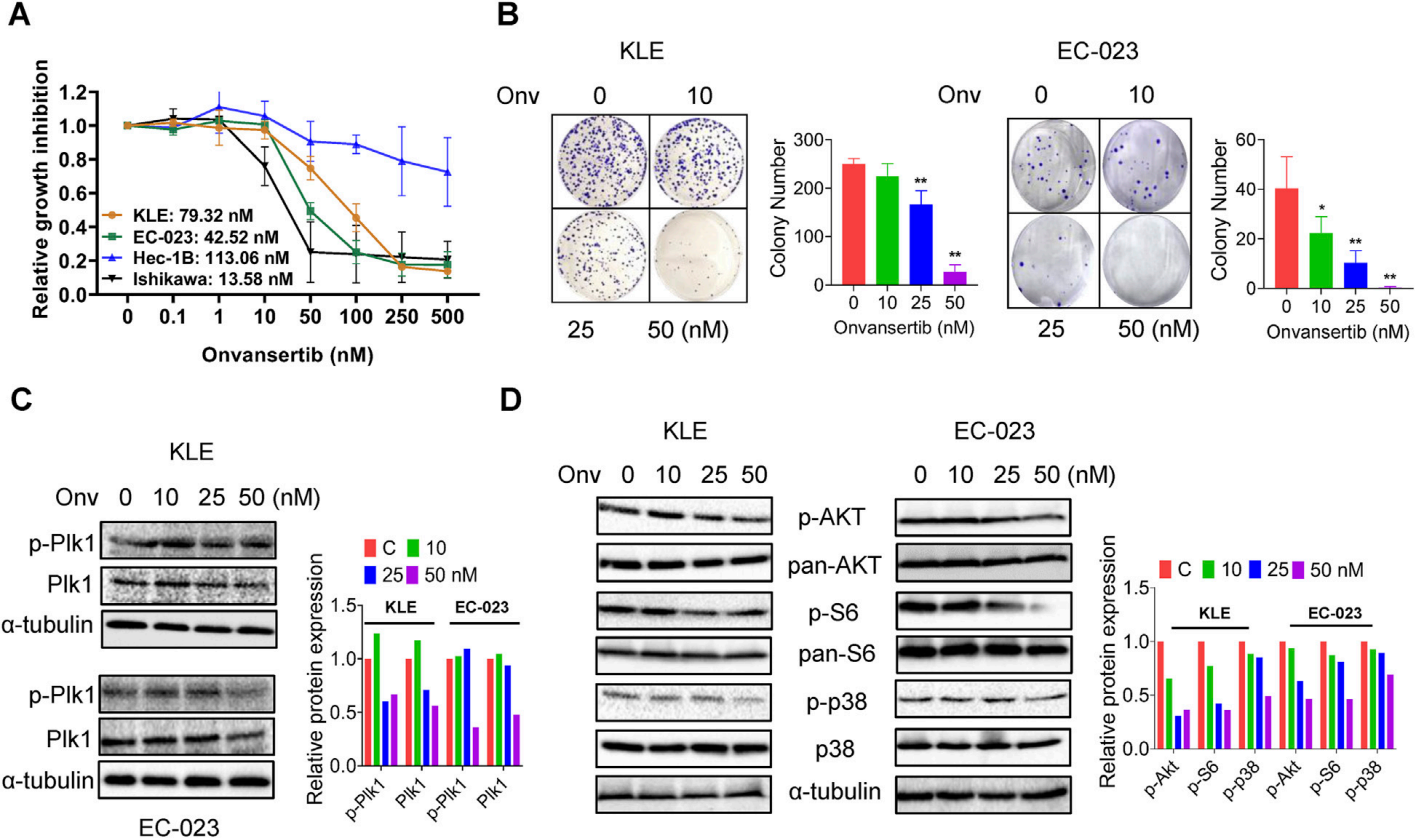
Effect of onvansertib on cell proliferation in EC cells. The KLE, EC-023, HEC-1B, and Ishikawa cells were treated with onvansertib at 0.1–500 nM for 72 h. Cell proliferation was detected by MTT assay. Onvansertib inhibited the proliferation of the 3 cell lines in a dose-dependent manner **(A)**. The KLE and EC-023 cells were treated with 10, 25, and 50 nM of onvansertib for 48 h and continually cultured for 10–14 days. Onvansertib inhibited the colony formation in both cell lines **(B)**. Western blotting results showed that onvansertib inhibited the expression of phosphorylated Plk1 and Plk1in both cell lines **(C)**. Western blotting results showed that onvansertib inhibit the expression of phosphorylated AKT, phosphorylated S6, and phosphorylated p38 in both cell lines after treatment for 24 h **(D)**. *p < 0.05, **p < 0.01.

Four EC cell lines, KLE, EC-023, HEC-1B, and Ishikawa, were treated with onvansertib at 0.1–500 nM for 72 h. Cell proliferation was assessed using the MTT assay. Onvansertib inhibited cellular proliferation in a dose-dependent manner in all cell lines. The IC50 values of the KLE, EC-023, HEC-1B, and Ishikawa cells were 79.32, 42.52, 113.06, and 13.58 nM, respectively (Figure 1A). The KLE and EC-023 cells were used to conduct further experiments. Colony formation assays were performed to understand the long-term effect of onvansertib on cell growth. The KLE and EC-023 cells were treated with 10, 25, and 50 nM onvansertib for 48 h, and both cell lines were cultured for an additional 10–14 days. The results showed that colony formation was significantly inhibited by 25 and 50 nM onvansertib in both cell lines. Colony formation in the KLE and EC-023 cells was reduced by 89.02% and 99.38%, respectively, after exposure to 50 nM onvansertib (Figure 1B). Western blotting results confirmed that onvansertib inhibited the expression of phosphorylated Plk1 in both cells after 24 h of treatment, indicating that onvansertib effectively reduced Plk1 activity in EC cells (Figure 1C).

Given the role of the Akt/mTOR and MAPK pathway in Plk1 inhibiting cell proliferation, the effect of onvansertib on the AKT/mTOR and MAPK signaling pathway was evaluated by Western blot in the KLE and EC-023 cell lines. Treatment of both cells with increasing doses of onvansertib for 24 h markedly decreased the expression of phosphorylated AKT, phosphorylated S6, and phosphorylated p38 in both cell lines (Figure 1D). Together, these results demonstrated that onvansertib is an effective Plk1 inhibitor which can effectively inhibit cell proliferation possibly via AKT/mTOR/S6 and MAPK signaling pathway in EC cells.

### Effect of onvansertib on cell cycle progression in EC cells

**FIGURE 2:**
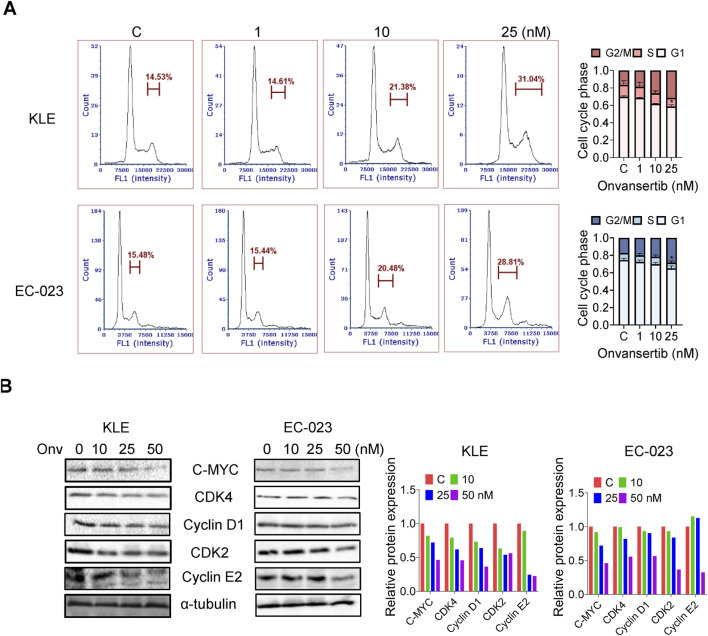
Effect of onvansertib on cell cycle progression in EC cells. The KLE and EC-023 cells were treated with 10, 25, and 50 nM of onvansertib for 24 h. The cell cycle profiles were assessed by Cellometer. Onvansertib caused cell cycle G2 phase arrest in both cell lines in a dose-dependent manner **(A)**. Western blotting results showed that onvansertib inhibited the expression of CDK4, cyclin D1, CDK2, cyclin E2 as well as C-MYC after treatment for 24 h in both cell lines **(B)**. *p < 0.05, **p < 0.01.

To understand the effect of onvansertib on cell cycle progression in the KLE and EC-023 cells, the cell cycle profile was analyzed by Cellometer after treating both cell lines with 10, 25, and 50 nM onvansertib for 24 h. Onvansertib reduced cell cycle G1 phase and increased G2 phase arrest in a dose-dependent fashion in both cell lines (Figure 2A). Treatment with 50 nM onvansertib increased the population of cells in the G2 phase from 14.5% to 31.6% and from 16.3% to 28.7% in KLE and EC-023 cells, respectively. Western blotting results showed that onvansertib significantly decreased the expression of phosphorylated PLK1 in both cell lines (Figure 2B). Furthermore, onvansertib effectively downregulated the protein expression of cell cycle-associated proteins CDK4, cyclin D1, CDK2, cyclin E2 as well as C-MYC in a dose-dependent fashion in both cell lines after treatment for 24 h (Figure 2B). These results confirmed that onvansertib effectively induced G2 phase arrest in KLE and EC-023 cells.

### Effect of onvansertib on cellular stress and apoptosis in EC cells

**FIGURE 3:**
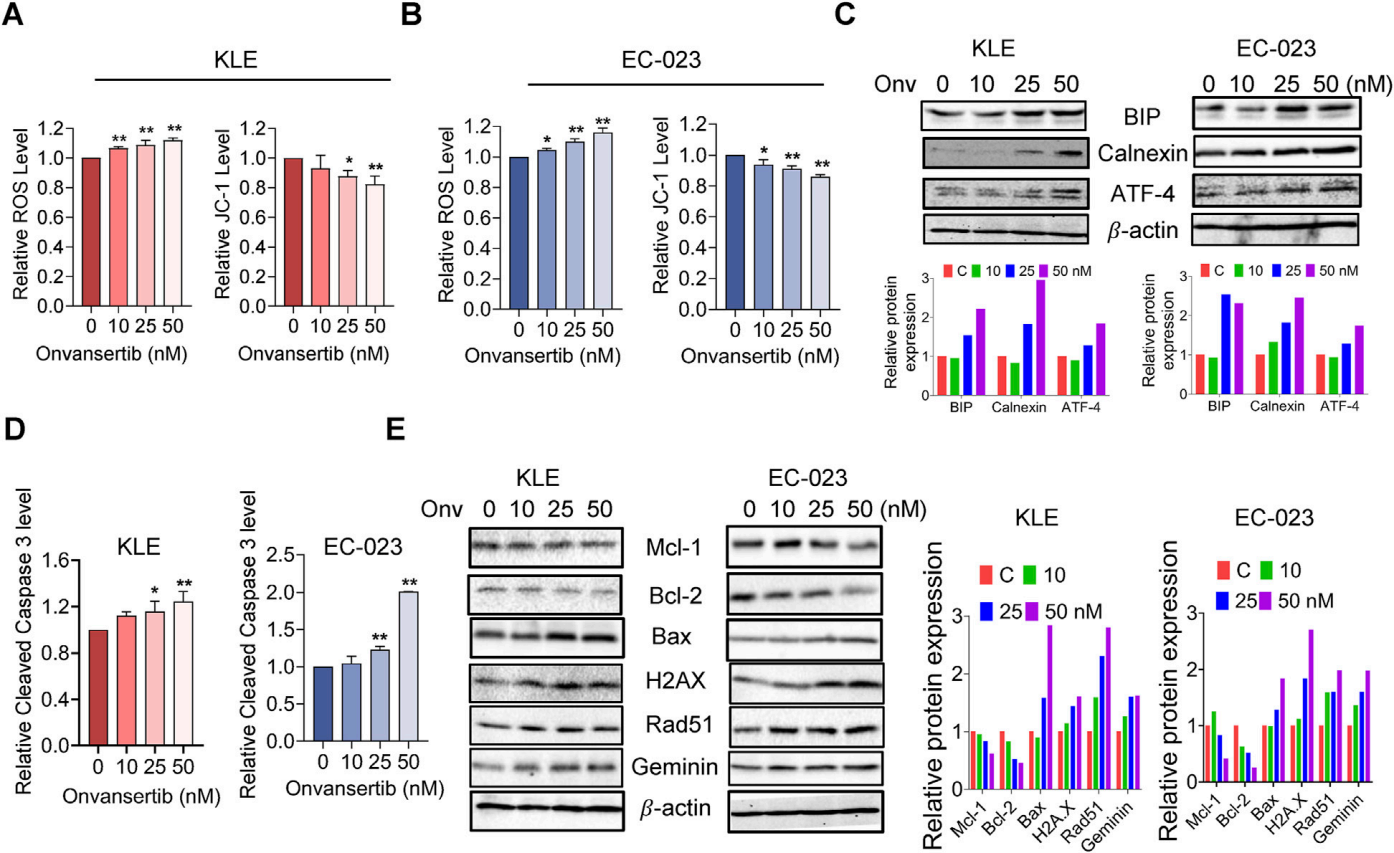
Effect of onvansertib on cellular stress and apoptosis in EC cells. The KLE and EC-023 cells were treated with 10, 25, and 50 nM of onvansertib for 6 h 25 and 50 nM Onvansertib increased the ROS levels, and decreased the JC-1 levels and in both cell lines **(A, B)**. Western blotting results demonstrated that onvansertib increased the expression of BiP, calnexin, and ATF-4 after 6 h of treatment in both cell lines, the double layer of ATF-4 is attribute to post-translational modifications **(C)**. ELISA assay was used to detect cleaved caspase three levels after treatment with 10, 25, and 50 nM of onvansertib for 14 h **(D)**. Western blotting results demonstrated that onvansertib increased the expression of BAX, H2AX, Rad51, and Geminin, while decreasing the expression of Mcl-1 and Bcl-2 after 24 h of treatment in both cell lines **(E)**. *p < 0.05, **p < 0.01.

Reactive oxygen species (ROS) have been known to play an integral role in the cellular response to stress and act as a mediator of apoptosis via mitochondrial DNA damage (Redza-Dutordoir and Averill-Bates, 2016). To investigate the effect of onvansertib on oxidative stress in EC cells, we utilized the DCHF-DA assay to assess intracellular ROS levels. Treatment with 10, 25, and 50 nM onvansertib for 6 h significantly increased cellular ROS production in a dose-dependent manner in the KLE and EC-023 cells (Figures 3A, B). With 50 nM onvansertib, ROS production was noted to increase by 11.8% in KLE cells and 15.9% in EC-023 cells, respectively, compared to control cells. JC-1 assay demonstrated that onvansertib reduced mitochondrial membrane potential with increasing doses of onvansertib in both cell lines. 50 nM onvansertib decreased JC-1 levels by 17.8% in KLE cells and 14.3% in EC-023 cells, respectively, compared with untreated cells (Figures 3A, B). Western blotting showed that onvansertib upregulated the expression of cellular stress-related proteins BiP, Calnexin, and ATF4 in both cell lines after 6 h of treatment (Figure 3C). These results suggest that onvansertib effectively induces cellular stress in EC cells.

To evaluate whether onvansertib causes apoptosis in EC cells, cleaved caspase three level was detected using an ELISA assay in both cell lines after treatment with 10, 25, and 50 nM onvansertib for 14 h. The results showed that both 25 nM and 50 nM onvansertib significantly increased cleaved caspase-3 levels when compared with control cells. 50 nM onvansertib increased the activity of cleaved caspase three to 1.24-fold in KLE cells and by 2.07-fold in EC-023 cells compared with the control cells (Figure 3D). Furthermore, Western blotting results showed that onvansertib downregulated the expression of the anti-apoptotic proteins Mcl-1 and Bcl-2, while upregulating the pro-apoptotic protein Bax in both cell lines after 24 h of treatment (Figure 3E). Meanwhile, onvansertib also increased the expression of the DNA damage markers H2AX, Rad51, and Geminin in a dose-dependent manner in both cell lines (Figure 3E). These results suggested that onvansertib effectively activates apoptotic pathways and induces DNA damage in EC cells.

### Effect of onvansertib on adhesion and invasion in EC cells

**FIGURE 4:**
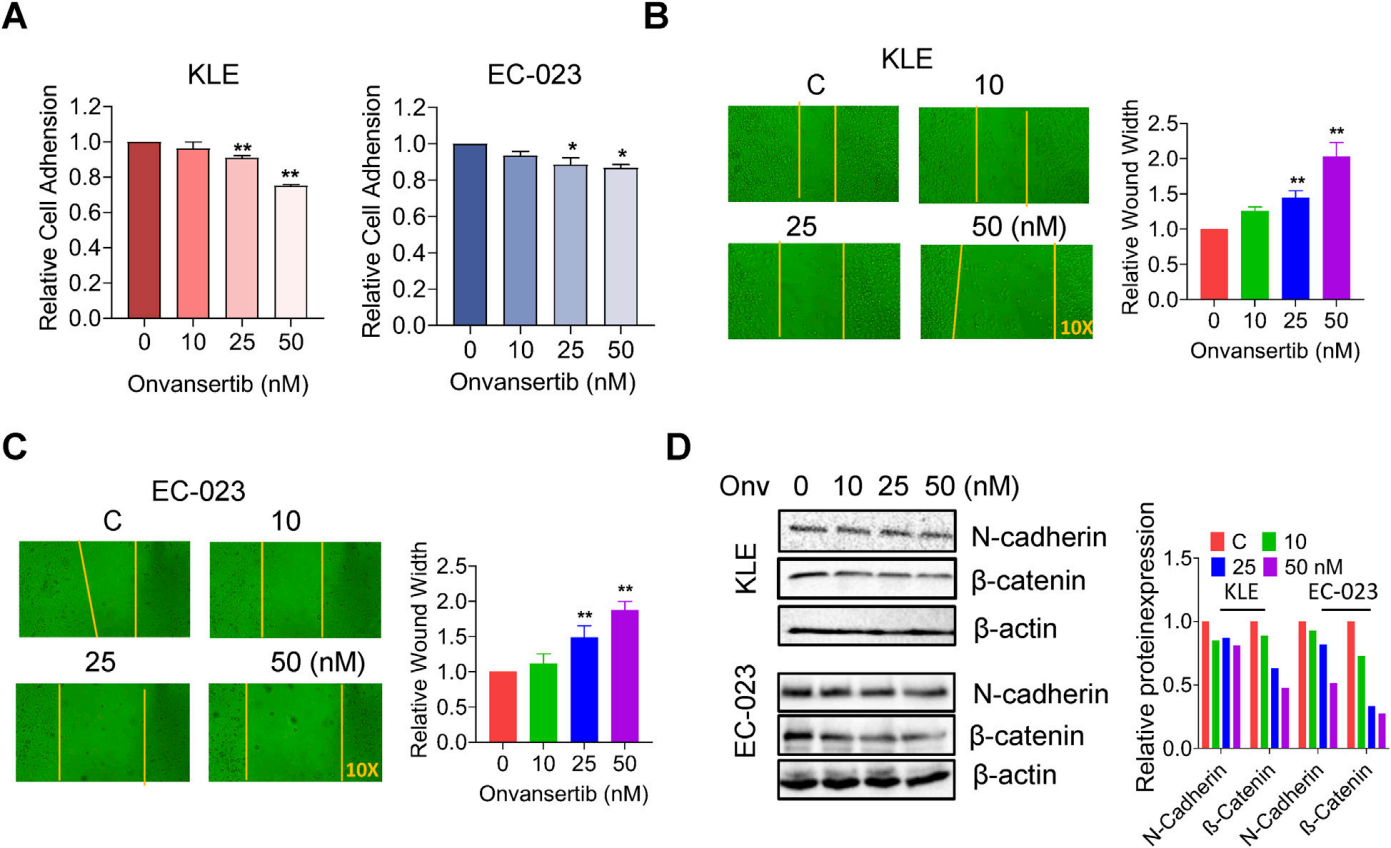
Effect of onvansertib on cellular adhesion and migration in EC cells. The effects of onvansertib on adhesion and migration were investigated by laminin-1 adhesion assay and wound healing assay, respectively. The laminin-1 assay showed that onvansertib significantly inhibited cell adhesion in both cell lines at 25 and 50 nM after 2 h of treatment **(A)**. The wound healing assay shows that treatment with 25 and 50 nM Onvansertib for 48 h resulted in wider wound widths, while the control group had narrower wound widths. The yellow line represents the boundary of wound healing **(B, C)**. The KLE and EC-023 cells were treated with 10, 25, and 50 nM of onvansertib for 24 h–48 h. Western blotting showed that onvansertib decreased the expression of β-catenin and N-cadherin after 24 h of treatment in both cell lines **(D)**. *p < 0.05, **p < 0.01.

We next investigated the effect of onvansertib on adhesion and invasion in EC cell lines. Using the laminin adhesion assay, we evaluated the impact of onvansertib on cell adhesion after treatment with 10, 25, and 50 nM onvansertib for 2 h. Cell adhesion was decreased by 24.84% and 13.29% in KLE and EC-023 cells, respectively, after treatment with 50 nM onvansertib Figure 4A. In a wound healing assay, cell migration was noted to be significantly reduced by 25 and 50 nM onvansertib in both cell lines after 48 h of treatment, with 50 nM onvansertib increasing the wound width to 1.96-fold and 1.85-fold in the KLE and EC-023 cells, respectively (Figures 4B, C). Furthermore, Western blot confirmed that 10, 25, and 50 nM onvansertib significantly decreased the expression of N-cadherin and β-catenin in both cell lines after 24 h of treatment (Figure 4D). Taken together, these results indicate that inhibition of cell adhesion and invasion by onvansertib on cell invasion may be involved in the EMT pathway in EC cells.

### Onvansertib increased sensitivity to paclitaxel in EC cells

**FIGURE 5:**
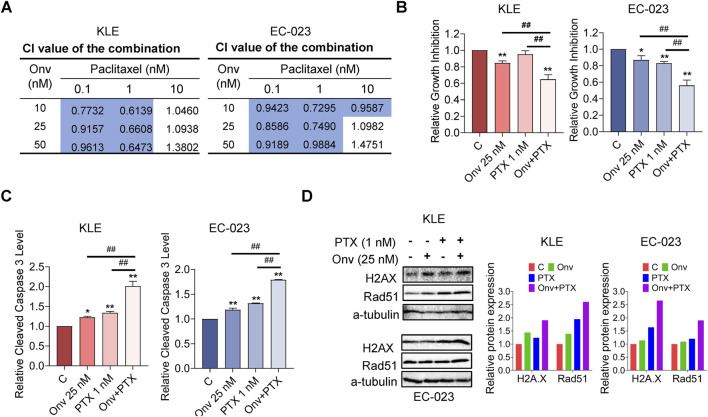
Onvansertib increased sensitivity to paclitaxel in EC cells. The KLE and EC-023 cells were each treated with 10, 25, and 50 nM of onvansertib, 0.1, 1, 10 nM of paclitaxel, and both in combination for 72 h. The Bliss independence model was used to calculate the combination index (CI) for each combination group **(A)**. The combination of 25 nM onvansertib and 1 nM paclitaxel resulted in greater cell growth inhibition than paclitaxel or onvansertib alone in both KLE and EC-023 cells after 72 h of treatment **(B)**. The combination of 25 nM onvansertib and 1 nM paclitaxel significantly increased cleaved caspase three levels compared with either paclitaxel or onvansertib alone after treatment for 14 h in both cell lines **(C)**. Both cell lines were treated with 25 nM onvansertib, 1 nM paclitaxel and the combination for 24 h. Western blotting showed that the combination group demonstrated a more potent effect on the expression of H2A.X and RAD51 compared with paclitaxel or onvansertib alone **(D)** *p < 0.05, **p < 0.01 compared with control. #p < 0.05, ##p < 0.01 compared with each group.

Given that the combination of onvansertib and paclitaxel (PTX) had been previously reported to have synergistic DNA damage response, we evaluated whether onvansertib could increase sensitivity to PTX. After treatment with three doses of onvansertib and PTX for 72 h, the MTT results showed that combination of onvansertib (10, 25, and 50 nM) and PTX (0.1 and 1.0 nM) exhibited potent synergy in inhibiting cell proliferation in both cell lines (Figure 5A, CI < 1). Treatment with 25 nM onvansertib plus 1 nM PTX led to 35.24% and 43.92% cell growth inhibition in the KLE and EC-023 cells respectively, whereas onvansertib alone and PTX alone produced 15.43% and 4.95% cell growth inhibition in KLE cells, and 13.05% and 17.02% in EC-023 cells, respectively (Figure 5B). This combination treatment also significantly increased apoptosis compared to onvansertib or PTX alone, as 25 nM onvansertib plus 1 nM PTX increased cleaved caspase three level to 2.01-fold and 1.79-fold in the KLE and EC-023 cells, respectively, whereas onvansertib alone and PTX alone elevated the cleaved caspase three level by 1.22-fold and 1.34-fold in the KLE and EC-023 cells, respectively (Figure 5C). Furthermore, Western blotting demonstrated that onvansertib increased the expression of DNA damage markers, including phosphorylated H2A.X and RAD51, after 24 h of treatment, while the combination treatment was more potent in upregulating the expression of phosphorylated H2A.X and RAD51 compared to onvansertib or PTX alone after 24 h of treatment in the KLE and EC-023 cells (Figure 5D). These results indicate that the combination of onvansertib and PTX synergistically reduces cell proliferation while inducing apoptosis and DNA damage.

### Onvansertib inhibited tumor growth in the *LKB1* ^*fl/fl*^ *p53* ^*fl/fl*^ transgenic mouse model of EC

**FIGURE 6:**
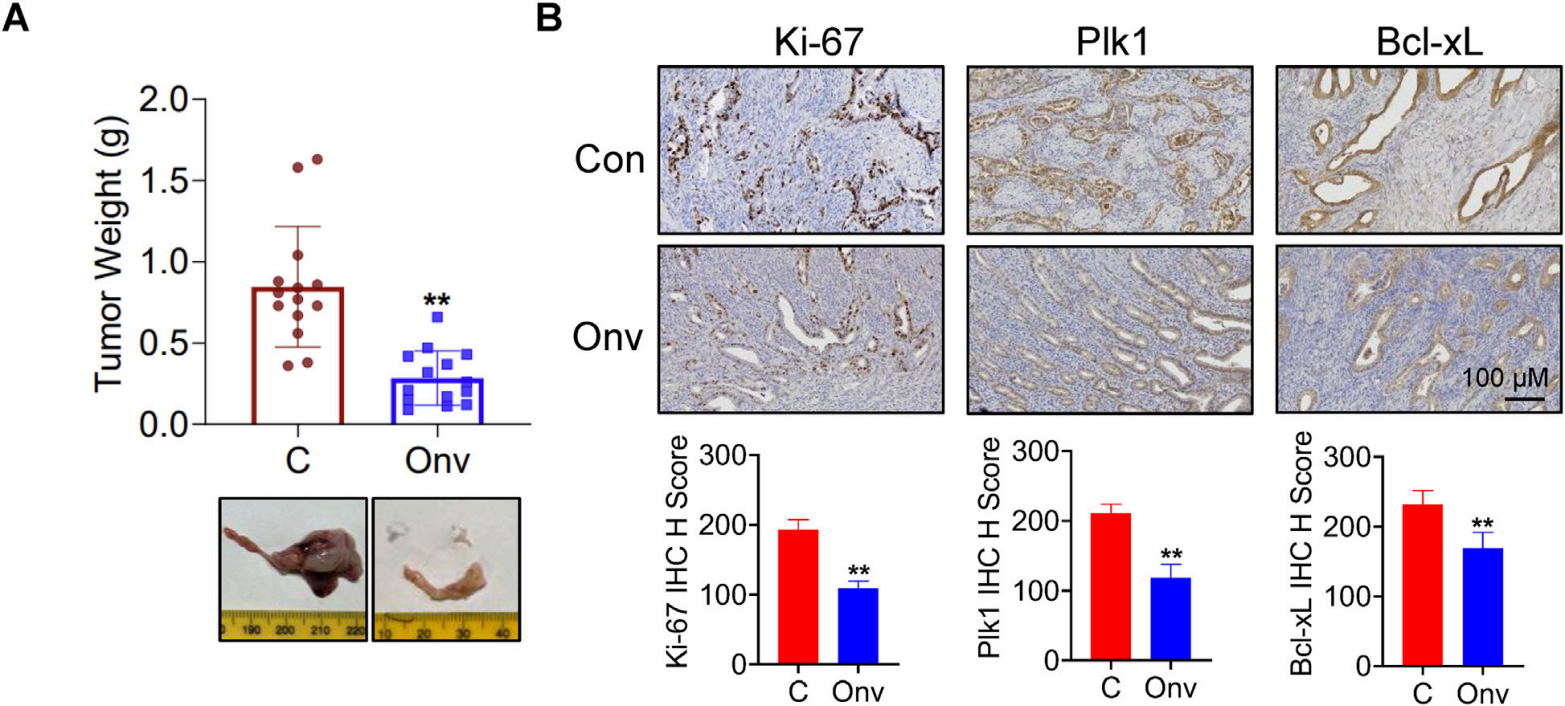
Onvansertib inhibits tumor growth in *LKB1* ^*fl/fl*^ *p53* ^*fl/fl*^ transgenic mouse model of EC. The *LKB1* ^*fl/fl*^ *p53* ^*fl/fl*^ genetically engineered mouse model of endometrioid endometrial cancer were treated with onvansertib (25 mg/kg, 100 µL per mouse for oral gavage, daily) or the vehicle (100 μL, 0.5% methylcellulose, daily) for 4 weeks. The effect of onvansertib on tumor weight in *LKB1* ^*fl/fl*^ *p53* ^*fl/fl*^ mice (n = 14) **(A)**. IHC staining results showed that onvansertib treatment significantly reduced the expression of Ki-67, Plk1, and Bcl-xL compared to the control mice (n = 6) **(B)**. *p < 0.05, **p < 0.01.

In order to better understand the role of onvansertib *in vivo*, the *LKB1* ^*fl/fl*^ *p53* ^*fl/fl*^ genetically engineered mouse model of endometrioid endometrial cancer was exposed to onvansertib. The mice were divided into two groups: onvansertib treatment and control groups (15 mice/group), and treated with onvansertib (25 mg/kg, 100 µL per mouse for oral gavage, daily) or the vehicle (100 μL 0.5% methylcellulose, daily) for 4 weeks. Tumor weights were significantly reduced by 66.94% in the onvansertib group compared to control group after 4 weeks of treatment (Figure 6A). During onvansertib treatment, mice showed normal activity and no significant changes in body weight (Data not shown). IHC staining results showed that compared with the control mice, onvansertib treatment significantly reduced the expression of Ki-67 by 44.01%, Plk1 expression by 40.6%, and Bcl-xL expression by 27.23%.in endometrial tumors (Figure 6B). Overall, these results confirmed that onvansertib effectively inhibits cell proliferation and tumor growth in EC cell lines and the transgenic mouse model of EC. Although our doses were selected based on literature-supported efficacy, dose optimization may be necessary in clinical trials to determine the optimal therapeutic window and balance efficacy and toxicity.

## Discussion

The success of CDK4/6 inhibitors in the treatment of patients with breast cancer has sparked the creation of novel cell cycle inhibitors that target various stages of the cell cycle, resulting in the emergence of various types of inhibitors with diverse anti-tumorigenic mechanisms (Chiappa et al., 2022; Ploumaki et al., 2024). Given that onvansertib alone or in combination with other chemotherapeutic agents has shown promising activity in clinical trials and overexpression of Plk1 has been demonstrated in EC, it is logical to investigate the therapeutic effects of onvansertib alone or in combination with other chemotherapeutic agents on cell proliferation and tumor growth in pre-clinical models of EC. In this study, we found that onvansertib significantly inhibited cell proliferation, caused cell cycle G2 arrest and apoptosis, induced cellular stress, reduced cell invasion, and increased the sensitivity to paclitaxel in EC cell lines. Furthermore, onvansertib effectively reduced tumor growth in the transgenic *LKB1* ^*fl/fl*^ *p53* ^*fl/fl*^ mouse model of endometrioid EC, which was accompanied by a reduction in the protein expression of Ki-67 and Plk1 and an increase in the expression of Bcl-xL in tumor tissues. Overall, these results provide pre-clinical evidence supporting the potential benefit of onvansertib alone or in combination with paclitaxel as an anti-cancer agent in EC.

Cancer cells require relatively higher levels of reactive oxygen species (ROS) than normal cells to maintain their biological activity, making them more susceptible to oxidative stress (Nelson et al., 2023; Liou and Storz, 2010). Excessive ROS levels trigger apoptotic signals in cancer cells by damaging various biomolecules beyond DNA repair (Liou and Storz, 2010). Plk1 is involved in inositol-requiring protein 1a (IRE1a, a cell stress sensor)-mediated induction of apoptosis and inhibition of proliferation in response to ER stress in cancer cells (Li et al., 2012). A certain intracellular ROS level is required for Plk1 to exert its biological functions, and inhibiting the production of ROS can partially change the anti-proliferative activity of Plk1 in cancer cells (Gao et al., 2024). Inhibiting Plk1 with BI2356 or volasertib significantly increased ROS levels in KRAS-mutant colon cancer, pancreatic cancer, and cervical cancer cells, while pre-treatment with a ROS scavenger partially reversed volasertib-induced apoptosis (Gao et al., 2024; Xin et al., 2019; Xie et al., 2015). Our results show that treatment of EC cells with onvansertib significantly increases ROS levels and decreases mitochondrial membrane potential, in parallel with increased expression of cellular stress proteins. Moreover, onvansertib also increased cleaved caspase three levels and reduced the expression of Mcl-1 and Bcl-2 in both cell lines. These findings suggest that inhibition of Plk1 by onvansertib induces cellular stress and apoptotic pathways, leading to the inhibition of cell proliferation in EC cells.

Myometrial invasion and lympho-vascular space invasion by EC cells are important factors affecting the metastatic spread of EC (38). Plk1 and its downstream targets, including mitotic spindle positioning (MISP), RhoGDI1, and FoxM1, regulate proliferative and invasive capabilities through epithelial-mesenchymal transition (EMT) transition in cancer cells (Lim et al., 2024; Pan et al., 2024; Xu et al., 2023). Increased Plk1 expression effectively promotes invasive ability in breast, renal, cervical, and thyroid cancer cells (Fu and Wen, 2017; Lim et al., 2024). Downregulation of Plk1 by small molecular inhibitors or siRNA significantly reduces cell invasion and migration in multiple types of cancer cells (Song et al., 2018; Fu and Wen, 2017; Zhou et al., 2020). Our results showed that onvansertib treatment reduces adhesion and migration while reducing N-cadherin and β-catenin expression in EC cells. The mechanisms underlying the effects of Plk1on adhesion and invasion of EC need to be addressed in future studies.

PTEN deletion and PI3K mutations as well as abnormal PI3K/AKT pathway activity are the most common molecular events in the carcinogenesis and progression of EC (43). Plk1 has been shown to increase the phosphorylation of PTEN Ser-380, Thr-382, and Thr-383 sites, leading to PTEN functional inactivation and activation of the PI3K/AKT signaling pathway (Choi et al., 2014; Li et al., 2014). Multiple agents targeting the AKT/mTOR pathway have been investigated in pre-clinical models and clinical trials in EC and have shown promising results (Leskela et al., 2019; Lengyel et al., 2020; Heudel et al., 2022). Plk1 directly binds and phosphates MTORC1 component RPTOR/RAPTOR, thereby leading to inhibition of MTORC1 activity in interphase cells (Ruf et al., 2017). Inhibition of Plk1 activity by the Plk1 inhibitors volasertib effectively reduced the phosphorylation of AKT, mTOR and S6 in leukemia and Burkitt lymphoma cells (Choi et al., 2023; Chen and Pei, 2020). Onvansertib, in combination with the PI3Kα inhibitor alpelisib, has been shown to synergistically inhibit cell viability and suppress PI3K signaling in PIK3CA-mutant HR + breast cancer both *in vitro* and *in vivo* (Sreekumar et al., 2024). Similarly, the combination of AKT inhibitor ipatasertib and onvansertib also demonstrated a synergistic anti-tumor effect in prostate cancer (Nouri et al., 2024). Our results showed that onvansertib decreased the expression of phosphorylated AKT and S6 in both cell lines, suggesting that the interaction of Plk1 with the PTEN/PI3K/AKT/mTOR pathway is involved in the anti-proliferative activity of onvansertib in EC cells. Collectively, the current data supports a critical role of the PI3K/AKT pathway in mediating onvansertib’s function and its potential for combination strategies targeting this signaling further supporting its potential for combination strategies targeting this signaling pathway.

Elevated Plk1 activity is necessary for repair of double-strand breaks, and depletion of Plk1 is able to cause DNA damage. The mitotic arrest caused by Plk1 inhibition is at least partially due to the presence of unrepaired double-strand breaks in mitosis in cancer cells (Chabalier-Taste et al., 2016; Driscoll et al., 2014). Thus, combining Plk1 inhibitors with DNA-damaging agents may be an ideal option to improve clinical efficacy (Chabalier-Taste et al., 2016; Rödel et al., 2010). Onvansertib treatment has been shown to increase radiosensitivity of MYC-driven medulloblastoma *in vitro* and *in vivo* (Wang et al., 2022). Furthermore, the combination of onvansertib and paclitaxel synergistically increased tumor growth in orthotopically transplanted PDXs of platinum-resistant ovarian carcinomas and showed a greater ability to induce γH2AX expression in tumor tissues (Affatato et al., 2022). In the present study, we treated KLE and EC-023 cells with onvansertib, paclitaxel, and their combination to study the synergistic effect of the combination in inhibiting cell growth. Our data showed that onvansertib effectively induced DNA damage and the combination of onvansertib and paclitaxel produced a synergistic effect in the inhibition of cell proliferation, which was accompanied by increased expression of γH2AX and Rad51 compared with paclitaxel or onvansertib alone in EC cells. Therefore, these results suggest that onvansertib combined with paclitaxel may be a good strategy for the treatment of recurrent EC and deserves further investigation in our transgenic mouse EC model. It is important to note that paclitaxel is a common first and second-line agent in the treatment of EC.

Several Plk1 inhibitors are currently being evaluated in clinical trials, both alone and in combination with other chemotherapy agents. Early phase I results indicated that onvansertib is well tolerated and biologically active in the treatment of advanced solid tumors (Ahn et al., 2024; Weiss et al., 2018). In multiple phase I/II clinical trials, onvansertib in combination with chemotherapy has shown significant anti-tumor activity in acute myeloid leukemia, colon cancer, among others (Su et al., 2022; Ahn et al., 2024; Zeidan et al., 2020). The commonly reported side effects of each drug (hematologic toxicity for onvansertib and neurotoxicity for paclitaxel) are also not overlapping, further supporting the continued investigation of this drug combination (Affatato et al., 2022; Weiss et al., 2018). Additionally, compared with other Plk1 inhibitors, onvansertib has oral bioavailability and a shorter half-life, and this allows optimization of the therapeutic window while minimizing toxicity when used with other chemotherapeutic agents including paclitaxel (Croucher et al., 2023). Considering changes in certain genetic alternations such as PTEN, p53, and KRAS have been shown to influence the sensitivity of cancer cells to onvansertib in preclinical models and some clinical trials, the identification of biomarkers that affect the sensitivity of EC to onvansertib in our subsequent studies is crucial to improve patient selection and enhance clinical treatment outcomes (Stebbing and Bullock, 2024b; Degenhardt et al., 2010).

## Conclusion

**FIGURE 7:**
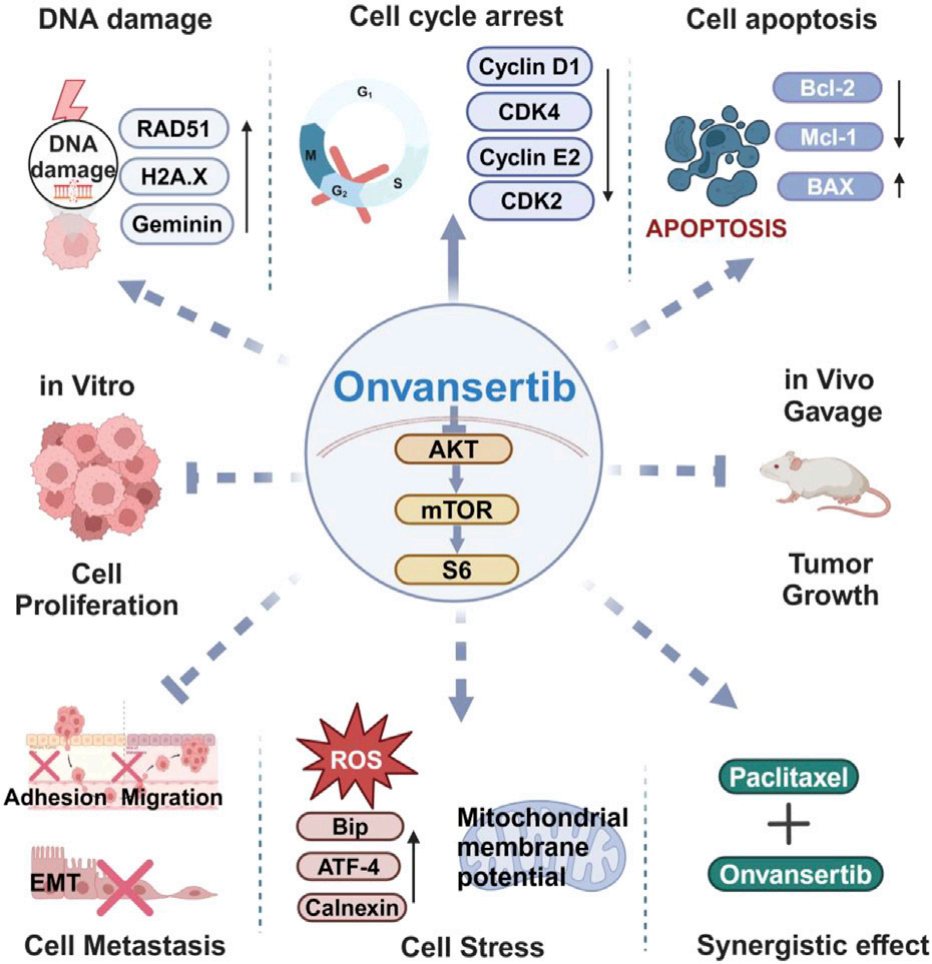
Graphical summary of the antitumor mechanisms of onvansertib in EC. Onvansertib effectively inhibited cell proliferation, caused cell cycle arrest, induced apoptosis and cell stress, reduced invasion ability, increased cell damage, and synergistically enhanced the sensitivity of EC cell lines to paclitaxel. Furthermore, treatment with onvansertib for 4 weeks significantly reduced tumor growth in the *LKB1* ^*fl/fl*^ *p53* ^*fl/fl*^ genetically engineered mouse model of endometrioid endometrial cancer. Solid arrow: Solid arrows: direct effect; dotted arrows: may act through cell cycle arrest.

In summary, our pre-clinical data found that targeting Plk1 by onvansertib demonstrated great potency in inhibiting cellular proliferation, inducing G2 cell cycle arrest and apoptosis, increasing cellular stress, impairing cellular adhesion, and reducing tumor growth in EC cells and the *LKB1* ^*fl/fl*^ *p53* ^*fl/fl*^ mouse model of endometrioid EC. Onvansertib combined with paclitaxel produced a synergistic effect of reducing cellular viability and increasing DNA damage in EC cells (Figure 7). Although this study has some limitations, including the short duration of *in vivo* experiments and the lack of long-term toxicity and resistance assessment in mice, our *in vitro* and *in vivo* results provide strong support and rationale for investigating the clinical translatability of onvansertib combined with paclitaxel as an effective treatment for endometrioid EC.

## Data Availability

The original contributions presented in the study are included in the article/supplementary material, further inquiries can be directed to the corresponding authors.

## References

[B1] Affatato R. Carrassa L. Chilà R. Lupi M. Restelli V. Damia G. (2020). Identification of PLK1 as a new therapeutic target in mucinous ovarian carcinoma. Cancers (Basel) 12 (3), 672. 10.3390/cancers12030672 32183025 PMC7140026

[B2] Affatato R. Chiappa M. Guffanti F. Ricci F. Formenti L. Fruscio R. (2022). Onvansertib and paclitaxel combined in platinum-resistant ovarian carcinomas. Ther. Adv. Med. Oncol. 14, 17588359221095064. 10.1177/17588359221095064 35665077 PMC9160919

[B3] Ahn D. H. Barzi A. Ridinger M. Samuëlsz E. Subramanian R. A. Croucher P. J. P. (2024). Onvansertib in combination with folfiri and bevacizumab in second-line treatment of KRAS-mutant metastatic colorectal cancer: a phase ib clinical study. Clin. Cancer Res. 30, 2039–2047. 10.1158/1078-0432.CCR-23-3053 38231047 PMC11094418

[B4] Cao S. Y. Fan Y. Zhang Y. F. Ruan J. Y. Mu Y. Li J. K. (2023). Recurrence and survival of patients with stage III endometrial cancer after radical surgery followed by adjuvant chemo- or chemoradiotherapy: a systematic review and meta-analysis. BMC Cancer 23 (1), 31. 10.1186/s12885-022-10482-x 36624407 PMC9827697

[B5] Chabalier-Taste C. Brichese L. Racca C. Canitrot Y. Calsou P. Larminat F. (2016). Polo-like kinase 1 mediates BRCA1 phosphorylation and recruitment at DNA double-strand breaks. Oncotarget 7 (3), 2269–2283. 10.18632/oncotarget.6825 26745677 PMC4823034

[B6] Chen E. Pei R. (2020). BI6727, a polo-like kinase 1 inhibitor with promising efficacy on Burkitt lymphoma cells. J. Int. Med. Res. 48 (5), 300060520926093. 10.1177/0300060520926093 32468878 PMC7263168

[B7] Chiappa M. Petrella S. Damia G. Broggini M. Guffanti F. Ricci F. (2022). Present and future perspective on PLK1 inhibition in cancer treatment. Front. Oncol. 12, 903016. 10.3389/fonc.2022.903016 35719948 PMC9201472

[B8] Choi B. H. Pagano M. Dai W. (2014). Plk1 protein phosphorylates phosphatase and tensin homolog (PTEN) and regulates its mitotic activity during the cell cycle. J. Biol. Chem. 289 (20), 14066–14074. 10.1074/jbc.M114.558155 24706748 PMC4022876

[B9] Choi E. J. Koo B. K. Hur E. H. Moon J. H. Kim J. Y. Park H. S. (2023). Inhibition of DNMT3B and PI3K/AKT/mTOR and ERK pathways as a novel mechanism of volasertib on hypomethylating agent-resistant cells. Biomol. Ther. Seoul. 31 (3), 319–329. 10.4062/biomolther.2022.117 36382510 PMC10129859

[B10] Croucher P. J. P. Ridinger M. Becker P. S. Lin T. L. Silberman S. L. Wang E. S. (2023). Spliceosome mutations are associated with clinical response in a phase 1b/2 study of the PLK1 inhibitor onvansertib in combination with decitabine in relapsed or refractory acute myeloid leukemia. Ann. Hematol. 102 (11), 3049–3059. 10.1007/s00277-023-05442-9 37702821 PMC10567832

[B11] Degenhardt Y. Greshock J. Laquerre S. Gilmartin A. G. Jing J. Richter M. (2010). Sensitivity of cancer cells to Plk1 inhibitor GSK461364A is associated with loss of p53 function and chromosome instability. Mol. Cancer Ther. 9 (7), 2079–2089. 10.1158/1535-7163.MCT-10-0095 20571075

[B12] Degenhardt Y. Lampkin T. (2010). Targeting Polo-like kinase in cancer therapy. Clin. Cancer Res. 16 (2), 384–389. 10.1158/1078-0432.CCR-09-1380 20068088

[B13] Driscoll D. L. Chakravarty A. Bowman D. Shinde V. Lasky K. Shi J. (2014). Plk1 inhibition causes post-mitotic DNA damage and senescence in a range of human tumor cell lines. PLoS One 9 (11), e111060. 10.1371/journal.pone.0111060 25365521 PMC4218841

[B14] Fu Z. Wen D. (2017). The emerging role of polo-like kinase 1 in epithelial-mesenchymal transition and tumor metastasis. Cancers (Basel) 9 (10), 131. 10.3390/cancers9100131 28953239 PMC5664070

[B15] Gao J. Huang W. Zhao S. Wang R. Wang Z. Ye J. (2024). Polo-like kinase 1 inhibitor NMS-P937 represses nasopharyngeal carcinoma progression via induction of mitotic abnormalities. J. Biochem. Mol. Toxicol. 38 (1), e23590. 10.1002/jbt.23590 38037286

[B16] Gheghiani L. Fu Z. (2023). The dark side of PLK1: implications for cancer and genomic instability. Oncotarget 14, 657–659. 10.18632/oncotarget.28456 37367493 PMC10295679

[B17] Guo H. Kong W. Zhang L. Han J. Clark L. H. Yin Y. (2019). Reversal of obesity-driven aggressiveness of endometrial cancer by metformin. Am. J. Cancer Res. 9 (10), 2170–2193. 31720081 PMC6834476

[B18] Hagege A. Ambrosetti D. Boyer J. Bozec A. Doyen J. Chamorey E. (2021). The Polo-like kinase 1 inhibitor onvansertib represents a relevant treatment for head and neck squamous cell carcinoma resistant to cisplatin and radiotherapy. Theranostics 11 (19), 9571–9586. 10.7150/thno.61711 34646387 PMC8490521

[B19] Heudel P. Frenel J. S. Dalban C. Bazan F. Joly F. Arnaud A. (2022). Safety and efficacy of the mTOR inhibitor, vistusertib, combined with anastrozole in patients with hormone receptor-positive recurrent or metastatic endometrial cancer: the VICTORIA multicenter, open-label, phase 1/2 randomized clinical trial. JAMA Oncol. 8 (7), 1001–1009. 10.1001/jamaoncol.2022.1047 35551299 PMC9100474

[B20] Karpel H. Slomovitz B. Coleman R. L. Pothuri B. (2023). Biomarker-driven therapy in endometrial cancer. Int. J. Gynecol. Cancer 33 (3), 343–350. 10.1136/ijgc-2022-003676 36878569

[B21] Kong W. Deng B. Shen X. John C. Haag J. Sinha N. (2024). Tirzepatide as an innovative treatment strategy in a pre-clinical model of obesity-driven endometrial cancer. Gynecol. Oncol. 191, 116–123. 10.1016/j.ygyno.2024.10.004 39388742 PMC12419173

[B22] Kuhn T. M. Dhanani S. Ahmad S. (2023). An overview of endometrial cancer with novel therapeutic strategies. Curr. Oncol. 30 (9), 7904–7919. 10.3390/curroncol30090574 37754489 PMC10528347

[B23] Lengyel C. G. Altuna S. C. Habeeb B. S. Trapani D. Khan S. Z. (2020). The potential of PI3K/AKT/mTOR signaling as a druggable target for endometrial and ovarian carcinomas. Curr. Drug Targets 21 (10), 946–961. 10.2174/1389450120666191120123612 31752654

[B24] Leskela S. Pérez-Mies B. Rosa-Rosa J. M. Cristobal E. Biscuola M. Palacios-Berraquero M. L. (2019). Molecular basis of tumor heterogeneity in endometrial carcinosarcoma. Cancers (Basel) 11 (7), 964. 10.3390/cancers11070964 31324031 PMC6678708

[B25] Li X. Zhu H. Huang H. Jiang R. Zhao W. Liu Y. (2012). Study on the effect of IRE1a on cell growth and apoptosis via modulation PLK1 in ER stress response. Mol. Cell Biochem. 365 (1-2), 99–108. 10.1007/s11010-012-1248-4 22314839

[B26] Li Z. Li J. Bi P. Lu Y. Burcham G. Elzey B. D. (2014). Plk1 phosphorylation of PTEN causes a tumor-promoting metabolic state. Mol. Cell Biol. 34 (19), 3642–3661. 10.1128/MCB.00814-14 25047839 PMC4187734

[B27] Lim J. Hwang Y. S. Yoon H. R. Yoo J. Yoon S. R. Jung H. (2024). PLK1 phosphorylates RhoGDI1 and promotes cancer cell migration and invasion. Cancer Cell Int. 24 (1), 73. 10.1186/s12935-024-03254-z 38355643 PMC10865702

[B28] Liou G. Y. Storz P. (2010). Reactive oxygen species in cancer. Free Radic. Res. 44 (5), 479–496. 10.3109/10715761003667554 20370557 PMC3880197

[B29] Liu Z. Sun Q. Wang X. (2017). PLK1, A potential target for cancer therapy. Transl. Oncol. 10 (1), 22–32. 10.1016/j.tranon.2016.10.003 27888710 PMC5124362

[B30] Matthess Y. Raab M. Knecht R. Becker S. Strebhardt K. (2014). Sequential Cdk1 and Plk1 phosphorylation of caspase-8 triggers apoptotic cell death during mitosis. Mol. Oncol. 8 (3), 596–608. 10.1016/j.molonc.2013.12.013 24484936 PMC5528627

[B31] Nelson V. K. Nuli M. V. Mastanaiah J. Saleem T. S. M. Birudala G. Jamous Y. F. (2023). Reactive oxygen species mediated apoptotic death of colon cancer cells: therapeutic potential of plant derived alkaloids. Front. Endocrinol. (Lausanne) 14, 1201198. 10.3389/fendo.2023.1201198 37560308 PMC10408138

[B32] Nouri M. Varkaris A. Ridinger M. Dalrymple S. L. Dennehy C. M. Isaacs J. T. (2024). AKT inhibition sensitizes to polo-like kinase 1 inhibitor onvansertib in prostate cancer. Mol. Cancer Ther. 23 (10), 1404–1417. 10.1158/1535-7163.MCT-23-0933 38894678 PMC11444904

[B33] Pan Y. R. Lai J. C. Huang W. K. Peng P. H. Jung S. M. Lin S. H. (2024). PLK1 and its substrate MISP facilitate intrahepatic cholangiocarcinoma progression by promoting lymphatic invasion and impairing E-cadherin adherens junctions. Cancer Gene Ther. 31 (2), 322–333. 10.1038/s41417-023-00705-z 38057358 PMC10874889

[B34] Ploumaki I. Triantafyllou E. Koumprentziotis I. A. Karampinos K. Drougkas K. Karavolias I. (2024). Cyclin-dependent kinase 4/6 inhibitors as neoadjuvant therapy of hormone receptor-positive/HER2-negative early breast cancer: what do we know so far? Clin. Breast Cancer 24 (3), e177–e185. 10.1016/j.clbc.2024.01.002 38320891

[B35] Rafiee A. Mohammadizadeh F. (2023). Association of lymphovascular space invasion (lvsi) with histological tumor grade and myometrial invasion in endometrial carcinoma: a review study. Adv. Biomed. Res. 12, 159. 10.4103/abr.abr_52_23 37564444 PMC10410422

[B36] Redza-Dutordoir M. Averill-Bates D. A. (2016). Activation of apoptosis signalling pathways by reactive oxygen species. Biochim. Biophys. Acta 1863 (12), 2977–2992. 10.1016/j.bbamcr.2016.09.012 27646922

[B37] Rödel F. Keppner S. Capalbo G. Bashary R. Kaufmann M. Rödel C. (2010). Polo-like kinase 1 as predictive marker and therapeutic target for radiotherapy in rectal cancer. Am. J. Pathol. 177 (2), 918–929. 10.2353/ajpath.2010.100040 20581060 PMC2913372

[B38] Ruf S. Heberle A. M. Langelaar-Makkinje M. Gelino S. Wilkinson D. Gerbeth C. (2017). PLK1 (polo like kinase 1) inhibits MTOR complex 1 and promotes autophagy. Autophagy 13 (3), 486–505. 10.1080/15548627.2016.1263781 28102733 PMC5361591

[B39] Siegel R. L. Giaquinto A. N. Jemal A. (2024). Cancer statistics, 2024. CA Cancer J. Clin. 74 (1), 12–49. 10.3322/caac.21820 38230766

[B40] Song R. Hou G. Yang J. Yuan J. Wang C. Chai T. (2018). Effects of PLK1 on proliferation, invasion and metastasis of gastric cancer cells through epithelial-mesenchymal transition. Oncol. Lett. 16 (5), 5739–5744. 10.3892/ol.2018.9406 30405751 PMC6202541

[B41] Sreekumar S. Montaudon E. Klein D. Gonzalez M. E. Painsec P. Derrien H. (2024). PLK1 inhibitor onvansertib enhances the efficacy of alpelisib in PIK3CA-mutated HR-positive breast cancer resistant to palbociclib and endocrine therapy: preclinical insights. Cancers (Basel) 16 (19), 3259. 10.3390/cancers16193259 39409880 PMC11476299

[B42] Stebbing J. Bullock A. J. (2024a). Polo-like kinase 1 inhibition in KRAS-mutated metastatic colorectal cancer. Clin. Cancer Res. 30, 2005–2007. 10.1158/1078-0432.CCR-24-0251 38470499

[B43] Stebbing J. Bullock A. J. (2024b). Polo-like kinase 1 inhibition in KRAS-mutated metastatic colorectal cancer. Clin. Cancer Res. 30 (10), 2005–2007. 10.1158/1078-0432.CCR-24-0251 38470499

[B44] Su S. Chhabra G. Singh C. K. Ndiaye M. A. Ahmad N. (2022). PLK1 inhibition-based combination therapies for cancer management. Transl. Oncol. 16, 101332. 10.1016/j.tranon.2021.101332 34973570 PMC8728518

[B45] Takai N. Miyazaki T. Fujisawa K. Nasu K. Hamanaka R. Miyakawa I. (2001). Polo-like kinase (PLK) expression in endometrial carcinoma. Cancer Lett. 169 (1), 41–49. 10.1016/s0304-3835(01)00522-5 11410324

[B46] Wang D. Veo B. Pierce A. Fosmire S. Madhavan K. Balakrishnan I. (2022). A novel PLK1 inhibitor onvansertib effectively sensitizes MYC-driven medulloblastoma to radiotherapy. Neuro Oncol. 24 (3), 414–426. 10.1093/neuonc/noab207 34477871 PMC8917408

[B47] Wang R. Hou Y. Geng G. Zhu X. Wang Z. Cai W. (2023). Onvansertib inhibits the proliferation and improves the cisplatin-resistance of lung adenocarcinoma via β-catenin/c-Myc signaling pathway. Am. J. Cancer Res. 13 (2), 623–637. 36895968 PMC9989612

[B48] Wei X. Song M. Huang C. Yu Q. Jiang G. Jin G. (2023). Effectiveness, safety and pharmacokinetics of Polo-like kinase 1 inhibitors in tumor therapy: a systematic review and meta-analysis. Front. Oncol. 13, 1062885. 10.3389/fonc.2023.1062885 36845678 PMC9947705

[B49] Weiss G. J. Jameson G. Von Hoff D. D. Valsasina B. Davite C. Di Giulio C. (2018). Phase I dose escalation study of NMS-1286937, an orally available Polo-Like Kinase 1 inhibitor, in patients with advanced or metastatic solid tumors. Invest New Drugs 36 (1), 85–95. 10.1007/s10637-017-0491-7 28726132

[B50] Weng Ng W. T. Shin J. S. Roberts T. L. Wang B. Lee C. S. (2016). Molecular interactions of polo-like kinase 1 in human cancers. J. Clin. Pathol. 69 (7), 557–562. 10.1136/jclinpath-2016-203656 26941182

[B51] Xie F. F. Pan S. S. Ou R. Y. Zheng Z. Z. Huang X. X. Jian M. T. (2015). Volasertib suppresses tumor growth and potentiates the activity of cisplatin in cervical cancer. Am. J. Cancer Res. 5 (12), 3548–3559. 26885445 PMC4731630

[B52] Xin X. Lin F. Wang Q. Yin L. Mahato R. I. (2019). ROS-responsive polymeric micelles for triggered simultaneous delivery of PLK1 inhibitor/miR-34a and effective synergistic therapy in pancreatic cancer. ACS Appl. Mater Interfaces 11 (16), 14647–14659. 10.1021/acsami.9b02756 30933478 PMC6712559

[B53] Xu R. Lee Y. J. Kim C. H. Min G. H. Kim Y. B. Park J. W. (2023). Invasive FoxM1 phosphorylated by PLK1 induces the polarization of tumor-associated macrophages to promote immune escape and metastasis, amplified by IFITM1. J. Exp. Clin. Cancer Res. 42 (1), 302. 10.1186/s13046-023-02872-1 37968723 PMC10652615

[B54] Zeidan A. M. Ridinger M. Lin T. L. Becker P. S. Schiller G. J. Patel P. A. (2020). A phase ib study of onvansertib, a novel oral PLK1 inhibitor, in combination therapy for patients with relapsed or refractory acute myeloid leukemia. Clin. Cancer Res. 26 (23), 6132–6140. 10.1158/1078-0432.CCR-20-2586 32998961

[B55] Zhao Z. Wang J. Kong W. Fang Z. Coleman M. F. Milne G. L. (2024a). Intermittent energy restriction inhibits tumor growth and enhances paclitaxel response in a transgenic mouse model of endometrial cancer. Gynecol. Oncol. 186, 126–136. 10.1016/j.ygyno.2024.04.012 38669767 PMC11216885

[B56] Zhao Z. Wang J. Kong W. Newton M. A. Burkett W. C. Sun W. (2024b). Palmitic acid exerts anti-tumorigenic activities by modulating cellular stress and lipid droplet formation in endometrial cancer. Biomolecules 14 (5), 601. 10.3390/biom14050601 38786008 PMC11117634

[B57] Zhou Y. Wu C. Liu B. Zhu J. Zhong Y. Yuan Y. (2020). shRNA targeting PLK1 inhibits the proliferation and invasion of nasopharyngeal carcinoma cells. Transl. Cancer Res. 9 (9), 5350–5359. 10.21037/tcr-20-811 35117900 PMC8798612

